# Circulating tumor DNA as a prognostic and monitoring biomarker in urothelial cancers

**DOI:** 10.3389/fonc.2026.1767712

**Published:** 2026-04-10

**Authors:** Jeanny B. Aragon-Ching, Fumihiko Urabe, Shugo Yajima

**Affiliations:** 1 Genitourinary (GU) Medical Oncology, Inova Schar Cancer Institute, Fairfax, VA, United States; 2 Department of Medical Education, University of Virginia, Charlottesville, VA, United States; 3 Department of Urology, The Jikei University School of Medicine, Tokyo, Japan; 4 Department of Urology, The Jikei University West Medical Center, Tokyo, Japan; 5 Department of Urology, National Cancer Center Hospital East, Kashiwa, Japan

**Keywords:** circulating tumor DNA (ctDNA), immune checkpoint inhibitors, locally advanced urothelial cancer, metastatic urothelial cancer, urothelial cancer

## Abstract

The application of circulating tumor DNA (ctDNA) has advanced in the treatment of multiple cancer types, including urothelial cancer (UC). While ctDNA currently plays a limited role in non-muscle-invasive bladder cancer (NMIBC), its relevance is expanding in muscle-invasive bladder cancer (MIBC), as evidenced by recent positive results from the IMvigor011 trial. This study demonstrated that a ctDNA-guided approach to adjuvant atezolizumab therapy enhances disease-free survival. Furthermore, ctDNA is well-established as a tool for monitoring treatment response and assessing minimal residual disease (MRD) in metastatic urothelial cancer (mUC). This narrative review explores the current data on the clinical utility of ctDNA in various disease states within UC to help guide clinicians in the appropriate setting and application of ctDNA based on retrospective, current, and emerging data.

## 1 Introduction

Bladder cancer ranks as the seventh most common cancer among men in the United States, with a higher incidence rate among men than in women (1). Globally, it is the ninth most prevalent cancer (2).

Circulating tumor DNA (ctDNA) plays a well-established role in certain solid tumors, including colorectal cancer, where a liquid biopsy assay has the ability to detect clinical recurrence that justifies the toxicity of additional adjuvant treatment in resected stage II cancers (3). However, its role in urothelial cancer (UC) is still emerging, although it is attractive given the known high mutation rates in bladder cancer and its ability to detect genomic alterations in metastatic patients (4). Currently, there is no universal consensus on ctDNA testing in UC, and efforts are underway to evaluate ctDNA in different settings (5). However, an increasing number of reports have shown the benefits of testing, especially in light of the recent positive results of the prospective trial IMvigor011, which showed that ctDNA-guided adjuvant atezolizumab improves outcomes when patients who test positive for ctDNA receive adjuvant therapy. Understanding of the current existing data will help clinicians recognize both the potential benefits and the limitations of obtaining ctDNA in clinical practice. In addition, ctDNA serves as a pragmatic, noninvasive liquid biopsy with applications in aspects of diagnosis, prognosis, surveillance, and treatment guidance (6) across various disease stages, including in non-muscle-invasive bladder cancer (NMIBC), muscle-invasive bladder cancer (MIBC), and in both localized and metastatic urothelial cancer (mUC) (7, 8).

The fundamental principle of ctDNA detection relies on the capability of the assay to detect DNA released into the bloodstream due to tumor cell death and secretion. It typically makes up a very small fraction (<1%) of the total circulating free DNA (cfDNA), depending on the tumor burden and cancer stage; hence, different yields are observed depending on different assays. Furthermore, ctDNA detection is contingent upon identifying specific mutations (e.g., point mutations, copy number changes, and microsatellite alterations) and epigenetic changes. There are also different host factors and assay issues. Detection is influenced by patient factors, such as age, body habitus, tumor factors (i.e., type, size, and stage), and technical issues. In addition, pre-analytical factors (e.g., blood collection methods, tube type, transport time, and environment) are crucial for accurate ctDNA measurement, especially since ctDNA levels are often low.

Studies on both plasma ctDNA and (urine tumor DNA, utDNA) abound and are under active investigation. However, despite the emerging available high-level data on urine ctDNA studies (9, 10), we have limited our narrative review to studies on the utility of ctDNA in bladder UCs.

The objective of this narrative review was to provide an outline of the value of ctDNA, different platforms, and studies in different settings of UC, along with the results and practical applications in the clinic. The following sections describe the studies that are impactful in the NMIBC, MIBC, and mUC settings.

## 2 ctDNA overview

Blood contains different cellular components, including cell-free DNAs, mRNAs, proteins, nucleic acids, and the non-encapsulated DNA that is obtained from a blood draw that is released from tumor cells as a result of apoptosis and is referred to as ctDNA (11). There are a few ways to perform ctDNA testing: one is a tumor-informed approach that utilizes patient-specific tissue sequencing to design plasma assays, such as the use of bespoke assays or the Signatera assay. Advantages include higher sensitivity and fewer false-positive results, but this approach often requires more tissue and can miss subclones. Another approach is tumor-agnostic or tumor-naive ctDNA testing, which assesses common cancer alterations without prior tissue probes and targets common hotspots, and can be an off-the-shelf testing (7). This tumor-naive approach is ideal for early detection and profiling and is beneficial for monitoring, especially when tissue is lacking or limited.

ctDNA assays are increasingly used in multiple other cancers and are increasingly applied in MIBC (12) and mUC to guide treatment decisions. However, there is no universal consensus on ctDNA testing in UC, and understanding its benefits and limitations is crucial for clinical application. Given its relatively short half-life of 30 min to 2 h, real-time monitoring of tumor-derived ctDNA is not only possible but can also be unreliable and is subject to variability, which can influence accuracy and reproducibility. Detection methods include droplet digital polymerase chain reaction (ddPCR) (13–15) and targeted next-generation sequencing (NGS) (for sensitive, mutation-focused testing), as well as whole-genome sequencing (16) and methylation profiling (to improve specificity when mutations are scarce), which are all associated with advantages and disadvantages (see **Table 1**).

**Table 1:** Circulating tumor DNA (ctDNA) assays.

Assay	Advantages/features	Reference
Next-generation sequencing	Limit of detection (LOD) for variant allele frequency (VAF) of 0.1%–11%	(5)
Droplet digital PCR (ddPCR)	Offers high sensitivity (LOD = 0.01%–0.1%), low cost, and rapid results	(15)
WGS-based MRD assays	Can detect ctDNA with an LOD as low as 0.0001%	(16)

*ctDNA*, circulating tumor DNA; *LOD*, limit of detection; *WGS*, whole-genome sequencing; *ddPCR*, droplet digital PCR; *MRD*, minimal residual disease.

## 3 Role of urine tumor DNA and urine biomarkers

As opposed to obtaining ctDNA, urine biomarkers, or urine tumor DNA (utDNA) are also a promising, noninvasive tool for molecular profiling in UC and are attractive since urine is in direct contact with the tumor in the bladder and contains both ctDNA and cell pellet DNA (cpDNA) from the shedding of tumor cells (17, 18). However, the accuracy of urinary ctDNA detection is heavily dependent on standardized collection and processing methods, as variability in sample handling can affect the results. There is still an ongoing debate regarding the methods for the analysis of whole urine *versus* the use of cell pellet and supernatant, as each contains different DNA sources. Technical challenges include extracting DNA from large urine volumes and maintaining its integrity, especially since utDNA is highly fragmented. However, cpDNA appears to be concordant with tumor tissue DNA (18). On the other hand, small comparative studies have shown utDNA to correlate more closely with tumor DNA than plasma ctDNA in a small number of 12 patients, where utDNA detected progression earlier (19). Large, multicenter studies have confirmed the diagnostic feasibility of utDNA assays, with sensitivity and specificity values often exceeding 75%–91% even for low-grade tumors (20, 21). UtDNA assays may outperform cytology or lessen the need for cystoscopy. Methylation assays, such as Urifind (22), the Bladder EpiCheck test (23, 24), and NGS to determine biomarkers, such as programmed death-ligand 1 (PD-L1) (25, 26), are some of the increasingly adopted urinary assays that could potentially aid in the diagnosis and assessment of disease clearance. Assays such as PredicineBEACON have detected utDNA clearance in patients who achieved a complete response after chemotherapy and immunotherapy (27), with long-term follow-up showing no recurrence among subjects with utDNA clearance, and patients who cleared their utDNA have been shown to have no recurrence (28). Therefore, utDNA has emerged as a technically feasible and clinically actionable biomarker for diagnosis, surveillance, and treatment monitoring in UC. While utDNA testing is poised to become an integral part of precision management in bladder cancer, more studies looking at the refinement and standardization of PCR, NGS, and methylation-based methods have yet to be conducted before it replaces urine cytology, ctDNA, or even cystoscopy for the early diagnosis of NMIBC or MIBC.

## 4 Clinical evidence and trials

A descriptive and narrative review was performed to evaluate the role of ctDNA in the NMIBC, MIBC, and mUC disease states, as discussed below.

### 4.1 Non-muscle-invasive bladder cancer

A few studies have evaluated ctDNA use in NMIBC (see **Table 2**). Although evidence is currently limited, ctDNA analysis has demonstrated prognostic value and presented potential utility in surveillance, which could be significant given that NMIBC is considered a curable disease. While discussion regarding the utility of utDNA was covered in the aforementioned section, studies on ctDNA have focused on early recurrence detection in NMIBC. One of the early studies by Hauser et al. assessed hypermethylated DNA in serum in all 227 participants (29), of whom 75 had NMIBC, and showed that the yield of methylated DNA was infrequent (<10%) and did not correlate with advanced bladder cancer cells, nor was it able to discriminate between bladder cancer *versus* non-bladder cancer cells. Using one to six personalized assays per patient, higher levels of tumor DNA correlated with disease progression (*p* = 0.032) (19). Plasma ctDNA in 82 patients with NMIBC showed correlation with larger tumors after performing transurethral bladder resection of tumor (TURBT) (30), which helped predict cystoscopy findings. A ddPCR assay (31) that assessed two different cohorts of patients [*n* = 363 with NMIBC and *n* = 468 undergoing radical cystectomy (RC)] that included urine ctDNA and plasma ctDNA showed that 36% of the patients with NMIBC harbored at least one *FGFR3* or *PIK3CA* mutation, suggesting another potential utility of these assays. In addition, this retrospective dataset, which included both urine and plasma ctDNA, showed the correlation of high tumor DNA levels in the plasma samples with recurrence, in particular in the cystectomy cohort (*p* = 0.016). In a small, retrospective cohort involving 23 patients with NMIBC (32), the ctDNA detection rate was approximately 35%. In a more contemporary cohort of 45 NMIBC patients (32) and 83 NMIBC patients, ctDNA detection preceded recurrence in 71% of the subjects, with a lead time of 137 days, and those who tested positive had a significantly higher risk of recurrence [hazard ratio (HR) = 5.4, 95%CI = 2.1–14.2]. Therefore, the search for an ideal assay for detection continues. While the use of ctDNA is promising in NMIBC, there are not enough studies confirming its utility in routine clinical practice, as it is unable to determine early progression that can be used in lieu of direct visualization.

**Table 2:** Summary of key non-muscle-invasive bladder cancer (NMIBC) trials on circulating tumor DNA (ctDNA).

Study/author	Method/assay	Cohort/patients	Main findings	Reference
Hauser et al.	Methylation analysis	75 NMIBC, 48 benign, 31 benign urologic, and 53 healthy	Identified bladder cancer with 62.1% sensitivity and 88.7% specificity; improved with larger tumors	(29)
Zhang et al.	Plasma ctDNA quantification	82 NMIBC (post-resection, BCG therapy)	Higher positivity in T1 *vs*. Ta tumors and lesions ≥3 cm; higher ctDNA correlated with shorter DFS and predicted BCG response	(30)
Christensen et al.	ddPCR for FGFR3/PIK3CA	363 NMIBC, 468 HR-NMIBC/MIBC (RC)	36% NMIBC and 11% RC patients had mutations; recurrence correlated with ctDNA levels; high ctDNA linked to recurrence (*p* = 0.016)	(31)
Birkenkamp-Demtröder et al.	Personalized ddPCR assay	22 tumors (12 NMIBC), 377 samples	ctDNA detected in 83% of cases; preceded progression by months in 67% of cases	(19)
Ben-David et al.	ctDNA pre-RC	NMIBC and MIBC patients	Undetectable ctDNA pre-RC associated with significantly improved oncological outcomes	(33)
Wang et al.	Tumor-informed ctDNA assay (Signatera^®^)	23 high-grade, mostly BCG-unresponsive NMIBC	ctDNA detected in 35% of cases; often preceded cystoscopic/imaging recurrence; enabled earlier restaging and escalation	(32)

*HR-NMIBC*, high-risk non-muscle-invasive bladder cancer; *MIBC*, muscle-invasive bladder cancer; *RC*, radical cystectomy; *DFS*, disease-free survival; *ddPCR*, droplet digital polymerase chain reaction; *FGFR3*, fibroblast growth factor receptor 3; *PIK3CA*, phosphatidylinositol-4,5-bisphosphate 3-kinase catalytic subunit alpha.

### 4.2 Muscle-invasive bladder cancer

Obtaining ctDNA in MIBC offers significant potential to inform prognosis, detect minimal or molecular residual disease (MRD), and guide treatment decisions (34) (see **Table 3**). Initial research has shown that ctDNA can reliably predict metastatic relapses in bladder cancer. Sfakianos et al. published a retrospective, real-world dataset that evaluated 167 patients and included 852 plasma samples from patients with MIBC before they underwent RC. The study revealed that patients with detectable ctDNA during MRD surveillance were associated with shorter disease-free survival (DFS) when compared with those patients with undetectable ctDNA (MRD: HR = 6.93, *p* < 0.001; surveillance: HR = 23.02, *p* < 0.001) (35). It was also revealed that patients who had undetectable ctDNA did not benefit from adjuvant therapy (*p* = 0.34). This established the foundational scientific rationale for obtaining ctDNA to guide therapeutic decisions. In a pre-RC cohort that included 135 patients (36), it was shown that 41 patients (30%) experienced a recurrence, alluding to the prognostic value of ctDNA, particularly in predicting recurrence. A pre-RC ctDNA-negative status was observed in 54 patients (40%). The recurrence-free survival (RFS) rates at 6, 12, and 21 months were 98%, 93%, and 82%, respectively. In addition, in 112 pre-RC patients for whom prospective tumor-informed ctDNA analysis was conducted before and after RC, it was shown that the detection rate of ctDNA before RC was 53% (in 59 patients) and was uniformly associated with poor RFS (log-rank *p* < 0.0001) (33), regardless of clinical tumor stage or receipt of neoadjuvant chemotherapy. Detectable ctDNA also predicts a higher risk of nodal disease [odds ratio (OR) = 5.4, 95%CI = 1.9–18.2, *p* = 0.003]. Furthermore, the persistently undetectable group had significantly better RFS than those subjects who had conversion (log-rank *p* < 0.001), with 12-month RFS rates of 97% *vs*. 51% and 18-month RFS rates of 88% *vs*. 51%, respectively, suggesting good prognostic value of an undetectable ctDNA. Post-cystectomy ctDNA status obtained at 4 months after RC was also utilized as an independent prognostic biomarker of tumor progression (HR = 5.290, *p* = 0.033) and cancer-specific survival (HR = 4.199, *p* = 0.038) (37). Ben-David et al. also studied 109 patients who had undergone RC and lymph node dissection. Prospectively collected serial longitudinal tumor-informed ctDNA analysis was performed for RFS. Patients with pathologic node-positive disease and undetectable pre- or post-cystectomy ctDNA status had similar RFS as patients with pN0 and undetectable ctDNA (38). Not surprisingly, patients with pathologic stage ≥pT3 or with pathologic node-positive disease (HR = 4.2, 95%CI = 1.43–12.3, *p* = 0.009) were found to be at risk of disease relapse. Another study that obtained ctDNA using ddPCR from 42 patients with MIBC showed that 24% of patients progressed at a median of 6 months (39), making it a valuable prognostic biomarker of tumor progression before RC (HR = 6.774) and at 4 months (HR = 3.673) and 12 months after RC (HR = 30.865, *p* < 0.05). More recently, a cohort that included both locally advanced UC and mUC patients also showed earlier prediction of radiographic relapse (40). However, the majority of these studies are all retrospective in nature, nevertheless already showing early signals of utility in prognostication with the use of ctDNA (41).

**Table 3:** Key trials using circulating tumor DNA (ctDNA) in muscle-invasive bladder cancer (MIBC).

Study/phase of trial (reference)	Patient group/setting	Key findings/outcomes	Hazard ratio (HR)/*p*-value	Comments
Sfakianos et al./retrospective (35)	*n* = 167 MIBC patients, pre/post-RC	Detectable ctDNA = shorter DFS; undetectable ctDNA = no benefit from adjuvant therapy	HR = 6.93 (MRD), HR = 23.02 (surveillance), *p* < 0.001; adjuvant therapy (*p* = 0.34)	852 plasma samples; adjuvant therapy not beneficial if ctDNA undetectable Limitations: non-randomized and retrospective
Ben-David et al./prospective cohort (36)	*n* = 135 MIBC patients, pre-RC	30% recurrence; undetectable ctDNA = better RFS at 6, 12, and 21 months	12-month RFS: 97% (undetectable) *vs.* 51% (conversion) (*p* < 0.001)	Undetectable ctDNA = favorable prognosis, may be a candidate for de-escalation strategies
Ben-David et al./prospective cohort (33)	*n* = 112 MIBC patients	ctDNA was detected before RC in 59 patients (53%) and was associated with poor RFS (log-rank *p* < 0.0001)	Higher risk of nodal disease (OR = 5.4, 95%CI = 1.9–18.2, *p* = 0.003)	Detectable ctDNA before definitive therapy with RC is predictive of nodal involvement, locally advanced disease, and disease recurrence.
Ben-David et al./prospective cohort (38)	*n* = 108 MIBC patients; s/p RC + extended LN dissection	RFS for pN0 (*n* = 353) and pN+ (*n* = 105) at 12, 24, and 36 months of 87% *vs*. 54%, 80% *vs*. 39%, and 74% *vs*. 35%, respectively	Log-rank *p* < 0.0001	Patients with pN+.after RARC had worse oncological outcomes than patients with pN0 disease.
Carrasco et al/retrospective (37)	*n* = 37 MIBC patients/4 months after RC	Independent prognostic biomarker for progression and cancer-specific survival	HR = 5.290 (progression), HR = 4.199 (survival)	Four mutations were analyzed in cell-free DNA to detect ctDNA.
Lindskrog et al/retrospective (41)	*n* = 68 MIBC patients/pre- and post-RC	ctDNA + sensitivity of 94% and specificity of 98%	ctDNA was a prognostic predictor before (HR = 3.4, *p* = 0.0005) and after RC (HR = 17.8, *p* = 0.0002).	ctDNA is prognostic in NAC-treated patients, and no difference in ctDNA− patients
Carrasco et al/retrospective (39)	*n* = 42 MIBC patients/pre-RC	24% of subjects progressed within 6 months; ctDNA was valuable before and after RC	HR = 6.774 (pre-RC), HR = 3.673 (4 months post-RC), HR = 30.865 (12 months post-RC) (*p* < 0.05)	Tumor-agnostic testing looking at two mutations using droplet digital PCR (*TERT* c.1-124C>T and *ATM* c.1236-2A>T)
IMvigor010 (phase III) (42)	*n* = 500 ypT2/pT3/pT4 or pN+; adjuvant atezolizumab *vs*. placebo	Baseline ctDNA+ = worse prognosis; ctDNA clearance = improved OS	37% of patients were ctDNA+; ctDNA+ improved OS in the atezolizumab arm (HR = 0.59)	Negative primary endpoint, but ctDNA status is informative
IMvigor011 (phase III) (49)	*n* = 405 ypT2/pT3/pT4 (if +NAC); adjuvant atezolizumab, ctDNA-guided	OS = 32.8 months (atezo) *vs*. 21.1 months (placebo); ctDNA-guided therapy improved OS	HR = 0.59 (death), 95%CI = 0.39–0.90, *p* = 0.01	Prospective confirmation of the ctDNA-guided benefit of adjuvant atezolizumab
CheckMate 274 (phase III *post-hoc*) (47)	*n* = 133 assessable MIBC patients; nivolumab *vs*. placebo	Baseline ctDNA detectable in 40.6% of patients; nivolumab improved DFS and OS in the ctDNA+ subgroup	mDFS = 52.1 months (95%CI = 19.4 months–NE), ctDNA undetectable *vs*. 5 months (95%CI = 2.8–6.5 months) ctDNA detectable at baseline (HR = 0.30, 95%CI = 0.18–0.48)	No benefit in ctDNA undetectable patients regardless of treatment
NIAGARA trial (phase III *post-hoc*) (48)	Peri-operative chemotherapy + durvalumab	ctDNA detectable rate: 57% baseline decreased to 22% post-neoadjuvant; ctDNA clearance = 41% in the durvalumab arm *vs*. 31% in chemotherapy	EFS improved in baseline ctDNA+ durvalumab *vs*. chemotherapy: HR = 0.73 (0.51–1.06)	ctDNA dynamics reported
ABACUS trial (phase II *post-hoc*) (52)	*n* = 40 MIBC patients/neoadjuvant atezolizumab pre-RC	ctDNA positivity: 63% baseline, 47% post-therapy, and 14% post-surgery	pCR = 31% (27/88; 95%CI = 21–41); 2-year DFS and OS were 68% (95%CI = 58–76) and 77% (95%CI = 68–85), respectively	Decline in ctDNA correlated with response
NABUCCO trial (phase Ib *post-hoc*) (53)	*n* = 30 stage III MIBC patients; preoperative nivolumab/ipilimumab	Absence of ctDNA correlated with pCR and PFS	Absence of plasma ctDNA correlated with pCR (OR = 45.0; 95%CI = 4.9–416.5) and PFS (HR = 10.4, 95%CI = 2.9–37.5)	ctDNA predictive of pCR in stage III UC
TOMBOLA (open-label phase II) (55)	*n* = 192 MIBC patients; NAC allowed; adjuvant atezolizumab post-RC	58% ctDNA+; median lead time 90 days over CT-detected metastasis; 1-year RFS = 97% in ctDNA− patients and 76% in ctDNA+ patients	63% of cases detected within 4 months post-RC; ctDNA dynamics correlated with RFS (*p* < 0.0001)	A Danish study showing that ctDNA+ identifies patients at high risk of relapse; ctDNA-negative patients showed excellent outcomes.

*RC*, radical cystectomy; *DFS*, disease-free survival; *RFS*, recurrence-free survival; *OS*, overall survival; *MRD*, minimal/molecular residual disease; *ICI*, immune checkpoint inhibitor; *pCR*, pathologic complete response; *HR*, hazard ratio; *OR*, odds ratio; *NE*, not estimable; *NAC*, neoadjuvant chemotherapy.

One of the notable early adjuvant immune checkpoint inhibitor (ICI) trials that informed much of our understanding of ctDNA dynamics is the phase III IMvigor010 trial (42), which did not meet its primary endpoint of achieving DFS after adjuvant atezolizumab compared with placebo in patients with a high risk of recurrence after RC. However, the study showed that baseline ctDNA positivity was associated with a poorer prognosis and that clearance of ctDNA correlated with improved overall survival (OS), especially among patients who received adjuvant atezolizumab (43, 44). These findings led to the prospective phase III trial that evaluated adjuvant atezolizumab, the IMvigor011 trial, which confirmed that ctDNA-guided therapy using adjuvant atezolizumab led to improvement in OS at 32.8 months *versus* an OS of 21.1 months in those subjects who had placebo (mortality: HR = 0.59, 95%CI = 0.39–0.90, *p* = 0.01) (45). *Post-hoc* exploratory ctDNA analyses from CheckMate 274 (46) were also presented at the European Society of Medical Oncology (ESMO) 2025, confirming that nivolumab improved DFS and OS by more than 2- and 1.5-fold, respectively, *versus* the placebo. Among the ctDNA-detectable subgroup of 54 out of 133 assessable patients who had baseline ctDNA detection after radical surgery (*n* = 54), the median DFS (mDFS) was 52.1 months (95%CI = 19.4 months–not estimable) in 79 patients *versus* 5.0 months (95%CI = 2.8–6.5 months) in patients with undetectable ctDNA (*n* = 54), with a HR of 0.30 (95%CI = 0.18–0.48). On the other hand, patients who had detectable ctDNA and were treated with nivolumab (*n* = 27) had an improved DFS of 91.9 months compared with those patients who had detectable ctDNA treated with a placebo (*n* = 27), who had an mDFS of 52.2 months with HR of 0.35 (95%CI = 0.18–0.66), while patients who already had undetectable ctDNA seemingly had no benefit regardless of nivolumab treatment (*n* = 50), with mDFS of 7.4 months *vs*. placebo (*n* = 29) with mDFS of 2.8 months (HR = 0.99, 95%CI = 0.51–1.93); however, the overall numbers were small (47). Serial ctDNA monitoring can provide valuable insights into the disease progression and inform treatment in mUC. In summary, ctDNA can be both prognostic in MIBC and predictive of the response to ICIs, although data are strongest in the prospective IMvigor011 trial that utilized atezolizumab. Another positive trial using perioperative chemotherapy with the ICI durvalumab, the NIAGARA trial (48), reported on ctDNA dynamics. A total of 462 patients were considered biomarker-evaluable, of whom the ctDNA detectable rate at baseline was 57% (260/460 patients), with a decline to 22% (94/422) after neoadjuvant treatment pre-RC (49). Another neoadjuvant trial utilizing the ICI durvalumab with tremelimumab and enfortumab vedotin, followed by cystectomy and then nine cycles of durvalumab in cisplatin-ineligible patients with MIBC, showed promising results (the VOLGA trial; NCT04960709) (50). The VOLGA trial utilized a cell-free DNA platform with GRAIL’s cancer research solution, which detects the methylation ctDNA status (GRAIL, LLC, Menlo Park, CA, USA). The ctDNA-positive rate was consistent with rates in other studies at 62.5% (10/16 patients), although the results are preliminary at the time of reporting. On the other hand, neoadjuvant atezolizumab prior to RC in the ABACUS trial showed ctDNA positivity values at baseline, after neoadjuvant therapy, and after surgery of 63% (25/40), 47% (14/30), and 14% (5/36), respectively (51). Updated ctDNA dynamic changes were shown, while ctDNA clearance was rare in 8% (3/40 patients). None relapsed. This ctDNA dynamic change is better than the assessment of ctDNA variant allele frequency (VAF) (52). Similarly, the absence of plasma ctDNA correlated with pathologic complete response (pCR) in preoperative nivolumab/ipilimumab in the NABUCCO trial (OR = 45.0, 95%CI = 4.9–416.5) and progression-free survival (PFS) (HR = 10.4, 95%CI = 2.9–37.5) (53). Another prospective trial, TOMBOLA (NCT04138628), analyzing the utility of adjuvant ICI post-RC, showed a 57% yield of ctDNA-positive findings as a preliminary result, with a median lead time of 43 days over metastatic disease observed on CT scans, with 75% being detected within 4 months post-RC (54). TOMBOLA was recently published, showing 1-year RFS and OS rates of 97% and 100%, respectively, in ctDNA-negative patients and only 76% and 88%, respectively, in ctDNA-positive patients, with a sensitivity of ctDNA approaching 92% (55). ctDNA was detected using patient-specific ddPCR, which found an 82.9% concordance with whole-genome sequencing (WGS) (56). Apart from the benefit of predicting recurrence, there is also the potential benefit of detecting *FGFR3* or *PIK3CA* mutations in both MIBC and NMIBC found in plasma ctDNA (31).

In summary, ctDNA demonstrates valuable prognostic and predictive utility for recurrence and OS in MIBC, with significant implications for guiding treatment strategies. Patients who are ctDNA-positive may benefit from adjuvant ICIs or other additional therapies, while patients who are ctDNA-negative could potentially avoid unnecessary toxicity, as their relapse-free survival is favorable regardless of additional treatment.

### 4.3 Metastatic urothelial cancer

Assessment of the ctDNA levels in mUC serves a broader purpose beyond prognostication and quantification (7). The utility of measuring MRD in mUC, particularly as it relates to treatment with ICIs to monitor treatment response and to guide treatment decisions, is key (17), especially given the molecular heterogeneity and progression to mUC (57). The use of ctDNA in mUC is particularly attractive given the potential shedding into the circulation, although it can be limited by sites of metastatic disease. The ctDNA dynamics from the failed Keynote 361 study, a phase III trial that explored pembrolizumab with and without chemotherapy, were retrospectively analyzed for ctDNA levels before and after treatment (58). Baseline ctDNA was linked to better overall response, PFS, and OS for pembrolizumab but not for chemotherapy. On the other hand, chemotherapy was found to result in a greater early quantitative decline in ctDNA levels; however, changes in ctDNA with pembrolizumab were more strongly associated with clinical outcomes. In addition, ctDNA metrics correlated with outcomes, but did not outperform standard imaging assessments using the RECIST criteria for predicting survival. ctDNA may correlate with disease burden and outcomes, and serial ctDNA testing tracks response and progression, but most importantly, ctDNA can reveal actionable alterations for targeted therapies (19). A systematic review and meta-analysis published by Ma and colleagues, which included a total of 862 patients from nine studies, showed that elevated baseline ctDNA levels were prognostic for shorter PFS/DFS (HR = 2.75, 95%CI = 1.36–5.58, *p* = 0.005) in subjects who were treated with ICIs, although not statistically significant. Early ctDNA dynamics and changes at week 8 helped predict response to ICI toripalimab (59) or to pembrolizumab based on the changes in VAF declines in *TP53* in patients with mUC (60). However, perhaps one of the best uses of ctDNA in mUC is its identification of targetable alterations, capitalizing on the generally good concordance between tissue and liquid biopsy (61–64). Since alterations in *FGFR* (65), *BRCA* (66), and *ERBB2* or *HER2* are targetable in mUC, it is all the more important to have a fairly dynamic liquid biopsy platform that enables clinicians to switch therapies whenever opportunities arise. Monitoring dynamic changes in ctDNA and measuring variations in the allele frequency changes predicted progression in a span of 6 months in approximately 90% of patients (67). BISCAY, an adaptive, biomarker-driven trial, combined durvalumab with anti-fibroblast growth factor receptor (FGFR) therapies to evaluate the correlation between circulating plasma-based DNA (ctDNA) and tissue for FGFR mutations, with changes over time in the ctDNA analyses correlating with outcomes (68).

## 5 Limitations

While multiple studies have utilized ctDNA in different disease sites, including NMIBC, MIBC, and metastatic stages, these studies are sometimes mixed with utDNA and profiling (69), which is beyond the scope of this review. The patient populations included in these analyses were heterogeneous, comprising both retrospective and prospective cohorts, which complicates the standardization of interpretation. Nevertheless, retrospective studies have contributed significantly to many earlier trials that established the rationale for the utility of ctDNA in UCs. While the majority of ctDNA assays are tumor-informed—particularly those utilizing the Signatera assay—this is not universally the case. Consequently, assay sensitivity may vary, and certain host factors may not be readily accounted for.

## 6 Future directions

Ongoing trials such as MODERN (clinicaltrials.gov NCT05987241) are evaluating the effectiveness of ctDNA-guided treatment strategies. IMvigor011 is among the major prospective trials integrating the use of ctDNA into the adjuvant treatment decision for MIBC, and has already shown early readout, justifying the incorporation of ctDNA into daily clinical practice and its impact on changing outcomes. However, as of this discussion, it has not yet been incorporated into major guidelines. The integration of ctDNA analysis with genetic, epigenetic, and multi-omics data may enhance MRD detection and inform therapy selection. Particularly in the mUC setting, optimal management of patients with positive or increasing ctDNA in the absence of radiologic evidence remains unresolved. Routine ctDNA testing is valuable in detecting mutations that can be targeted, although routine testing beyond this may not yield an additional advantage over radiologic imaging alone. The variability in assay sensitivity, the small sample sizes, and the lack of standardization across laboratories also present ongoing challenges. The clinical utility of ctDNA depends on the disease stage and the treatment context. Ultimately, robust randomized studies and extended follow-up are essential for its widespread adoption.

## 7 Conclusions

The integration of ctDNA testing and advancements in NGS has significantly transformed cancer diagnostics, prognostication, MRD monitoring, and therapeutic strategies. While the application of ctDNA analysis in NMIBC has shown considerable promise for surveillance and recurrence monitoring, the current evidence remains preliminary and does not yet support routine implementation in clinical practice. utDNA may have a promising yield in NMIBC, complementing assays such as cytology, urine biomarkers, and cystoscopy. On the other hand, the most promising results to date of ctDNA are in the MIBC field, especially given the positive findings of IMvigor011 showing that ctDNA-guided adjuvant therapy can alter outcomes in those patients who are ctDNA-positive and able to receive adjuvant atezolizumab. Given similar findings in CheckMate 274, one can perhaps extrapolate the results to any adjuvant ICI; however, prospective studies may be required to validate this. There have been numerous retrospective datasets suggesting benefits regarding prognostication using ctDNA, as observed in other trials that explored ctDNA dynamics, including IMvigor010 and ABACUS, which showed that post-surgery ctDNA positivity predicts poorer outcomes, while clearance of ctDNA during or after treatment correlates with better survival. Persistent ctDNA after surgery often precedes radiologic relapse, and methylation-based assays, such as the modified response (mR) score, may help predict chemotherapy response. In locally advanced and metastatic MIBC, ctDNA enables real-time mutation profiling, tracks disease progression, and helps identify biomarkers of response or resistance, such as *FGFR* and *ERBB2* alterations and *TP53*-related pathways. These insights support genotype-directed therapy and real-time monitoring, with the potential to reveal resistance mechanisms and guide treatment decisions, although the results can vary depending on the therapeutic strategy. ctDNA is a promising biomarker for prognosis and treatment guidance in UC. Its clinical utility is growing, but more validation is needed before routine adoption. Next-generation ctDNA assays integrating multi-omics may further improve outcomes.
